# Innovative methodologies for rare diseases clinical trials

**DOI:** 10.1186/s13023-024-03189-8

**Published:** 2024-05-07

**Authors:** Rima Nabbout, Ralf-Dieter Hilgers

**Affiliations:** 1 Reference centre for rare epilepsies Université Paris cité, Paris, France; 2 Assistance Publique-Hôpitaux de Paris, Hôpital Necker-Enfants Malades, Paris, France; 3 Institut Imagine, INSERM U1163, Paris, France; 4 RWTH Aachen University, Institute of Medical Statistics, 52074 Aachen, Germany

The European Joint Programme on Rare Diseases [1] brings over 130 institutions including all 24 European research networks (ERNs) from 35 countries:

- 26 EU Member States (Austria, Belgium, Bulgaria, Czech Republic, Denmark, Estonia, Finland, France, Germany, Greece, Hungary, Croatia, Ireland, Italy, Netherlands, Latvia, Lithuania, Luxembourg, Malta, Poland, Portugal, Romania, Spain, Sweden, Slovakia, Slovenia).
- 7 associated members (Armenia, Georgia, Israel, Norway, Serbia, Switzerland, Turkey)
- United Kingdom & Canada

aiming to create a comprehensive, sustainable ecosystem allowing a virtuous circle between research in rare diseases (RD), care and medical innovation. As clinical trials remain challenging in RD [2] even after regulatory recommendation [3], WP20 of EJP-RD aims to reach awareness of innovative trial methodology, not only limited to statistical methods. This is based on the previous developments of three FP7 funded projects (IDeAl [4], asterix [5] and InSpiRe [6]), which delivered a huge range on innovative statistical methods for small population clinical trials. However, these innovative methods need validation in practical settings. This gap is closed by EJP-RD WP20 launching demonstration projects, i.e. EPISTOP-IDeAl (EPISTOP clinical trial data set re-use with statistical methodologies tailored for clinical trials in rare diseases), EBStatMax (State-of-the-art statistical design and analysis for maximal success with minimal patient burden in Epidermolysis bullosa trials) and Improve PSP (Application of improved statistical methodologies for clinical trials in Progressive Supranuclear Palsy). To further address the need for innovations for limited population trials, WP20 funded also innovation calls in clinical trials methodologies for rare diseases i.e. iSTORE (Innovative statistical approaches to improve outcome in rare epilepsies: A model for rare and ultra-rare diseases) and Evidence RND (Creating robust evidence for longitudinal progression changes and treatment effects in ultra-rare neurological diseases: the case of multisystemic autosomal-recessive ataxias).

EBStatMax deals with State-of-the-art statistical design and analysis for maximal success with minimal patient burden in Epidermolysis bullosa trials, Improve PSP with application of improved statistical methodologies for clinical trials in Progressive Supranuclear Palsy and Epistop-IDeAl re-uses EPISTOP clinical trial data set on epilepsy preventive therapy in Tuberous sclerosis complex with statistical methodologies tailored for clinical trials in rare diseases.

The two innovation projects aim for improvement and innovation of the methodology for trials in limited populations, for example in relation to trial design, endpoints or the statistical analysis methods. For instance, iSTORE will develop Innovative Statistical Methodologies to Improve Rare Diseases Clinical Trials endpoints in Limited Populations using Dravet syndrome [7], a rare developmental and epileptic encephalopathy, as a show case [8]. EVIDENCE RND will create robust evidence for longitudinal progression changes and treatment effects in ultrarare neurological diseases: the case of multisystemic autosomal-recessive ataxias. The ambition is to reach results that might be transferrable to other rare diseases as well.

Beside these project-oriented developments, WP20 is driven by the mission to disseminate the developments and reach a higher level of trained rare disease researchers based on a survey organized by EJP-RD and having a dedicated part to clinical trials gaps and needs in the RD community. A core point are the advanced webinars; held by methodological experts and commented by a panel of experts from different stakeholders including in addition to methodologists, clinicians, patients representative, industry and members of the regulatory organisms. These webinars are recorded and made public and available at the EJP-RD site [9]. However and despite a high number of attendees, we realized that this has a limited outreach, and as science is disseminated usually by publications, WP20 was therefore very enthusiastic that Orphanet Journal of rare diseases welcomes to publish a series of papers dealing with these webinars and with the RD clinical trials developments. The unique series of publications allows linking the advanced webinars recordings and presentations with more insights on the topic and guarantees the ambition of reaching and training the largest rare disease scientific community.
